# Editorial: Big data and artificial intelligence in ophthalmology - clinical application and future exploration

**DOI:** 10.3389/fmed.2023.1339280

**Published:** 2023-12-06

**Authors:** Yong Yu Tan, Tyler Hyungtaek Rim, Darren S. J. Ting, Yi-Ting Hsieh, Tae-im Kim

**Affiliations:** ^1^Cork University Hospital, Cork, Ireland; ^2^Department of Ocular Epidemiology, Singapore Eye Research Institute, Singapore, Singapore; ^3^Mediwhale Inc., Seoul, Republic of Korea; ^4^Academic Unit of Ophthalmology, Institute of Inflammation and Ageing, University of Birmingham, Birmingham, United Kingdom; ^5^Birmingham and Midland Eye Centre, Birmingham, United Kingdom; ^6^Academic Ophthalmology, School of Medicine, University of Nottingham, Nottingham, United Kingdom; ^7^Department of Ophthalmology, National Taiwan University Hospital, Taipei, Taiwan; ^8^Department of Ophthalmology, College of Medicine, National Taiwan University, Taipei, Taiwan; ^9^Department of Ophthalmology, The Institute of Vision Research, Yonsei University College of Medicine, Seoul, Republic of Korea; ^10^Department of Ophthalmology, Corneal Dystrophy Research Institute, Yonsei University College of Medicine, Seoul, Republic of Korea

**Keywords:** big data, ophthalmology, artificial intelligence, deep learning, machine learning

## Introduction

Artificial Intelligence (AI) stands at the forefront of innovation in ophthalmology, harnessing vast datasets to redefine diagnostics and treatment strategies. This Research Topic collates pioneering insights from global experts, emphasizing AI's transformative impact on ophthalmic healthcare. Contributors have adeptly navigated the challenges, offering novel algorithms and applications poised to elevate patient care, streamline service delivery, and broaden healthcare access. From intricate retinal imaging to expansive electronic health record analyses, the papers within this topic not only underscore AI's potent capabilities but also chart a course for its future roles in enhancing ophthalmological practice.

## Diagnostic and predictive analytics

Won et al. presented a groundbreaking deep learning (DL) based classification system adept at distinguishing between bacterial and fungal keratitis through anterior segment photographs. Utilizing a dataset comprising 684 images from 107 patients confirmed with either bacterial or fungal keratitis, the study introduced two novel modules—the Lesion Guiding Module and the Mask Adjusting Module —which, when integrated with the ResNet-50 classifier, significantly outperformed the baseline model with an accuracy leap from 81.1 to 87.8%. The system's proficiency was further validated on an external set of 98 images, solidifying its potential as a rapid, reliable diagnostic tool in clinical settings.

Li et al. compared DL with human graders for evaluation against 300 fundus photographs. The AI's accuracy for diagnosing diabetic retinopathy and macular degeneration was on par with ophthalmologists, achieving an AUC of 0.990 and 0.945, respectively. It excelled in identifying glaucomatous optic neuropathy with an AUC of 0.994, better than human graders.

Liu et al. created ONION, a DL tool that discerns optic neuritis from optic neuropathy in acute phases, demonstrating an AUC of 0.903. Trained with EfficientNet-B0 on 871 eyes from 547 patients, ONION matched a retinal specialist's diagnostic ability, showing 79.6% sensitivity and 86.5% specificity in validation. It processed results in 23 s, highlighting its potential for rapid, accurate eye condition diagnosis in various healthcare settings.

Wang et al. introduced a novel DL approach to forecast postoperative visual outcomes in patients undergoing cataract surgery. Leveraging a dataset of 2051 eyes, their Model V achieved the lowest mean absolute error of 0.1250 and 0.1194 logMAR, and RMSE of 0.2284 and 0.2362 logMAR in the validation and test datasets, respectively. It achieved up to 91.7% precision and 93.8% sensitivity, indicating high predictive reliability and marking progress in preoperative patient assessments.

Shivananjaiah et al.'s study offerred a novel approach to predicting the likelihood of glaucoma progression to surgical intervention within 1 year, using DL. The researchers curated a cohort from electronic health records at Stanford University, capturing both structured data and free-text clinical notes from 2008 to 2020. The DL model was fed a blend of text embeddings from patient notes and structured clinical data, resulting in an impressive model performance—most notably, the multimodal fusion model exhibited an AUC of 0.899 and an F1 score of 0.745. This work demonstrates the potential role of DL in improving glaucoma treatment predictions using comprehensive patient data.

## Image analysis and segmentation

Imran et al. introduced Feature Preserving Mesh Network (FPM-Net), a network that segments retinal vasculature semantically without preprocessing, crucial for supporting the analysis of ophthalmic diseases, achieving exceptional accuracy (96.92% on DRIVE, 97.28% on CHASE-DB, 97.27% on STARE) and efficiency with only 2.45 million parameters. The research showcases FPM-Net's proficiency in preserving detailed spatial features for improved segmentation performance, essential for accurate retinal vessel analysis, making it a valuable tool for early diagnosis and management of ophthalmic diseases.

Bai et al. developed DME-DeepLabV3+ model, a lightweight and proficient model for the extraction of diabetic macular oedema (DME) from optical coherence tomography (OCT) images. This study harnesses the DeepLabV3+ architecture to address the complexity of OCT images, where varied image quality and the blurred boundaries of DME regions pose a significant challenge. Evaluated using a dataset of 1711 OCT images and validated by experienced clinicians, the DME-DeepLabV3+ achieves remarkable performance metrics, including a mean Intersection over Union (MIoU) of 91.18% and high precision and recall rates. This innovation promises to streamline the diagnostic process, offering a rapid, automated, and accurate tool for DME extraction.

## Innovation in disease detection and management

Gibson et al. harnessed the power of latent diffusion augmentation to enhance the DL analysis of neuro-morphology in limbal stem cell deficiency (LSCD). The study showcased a residual U-Net model, informed by the InceptionResNetV2 transfer learning model, to classify neuron morphology across various stages of LSCD compared to healthy controls. The model achieved accuracy in determining nerve fiber number (*R*-squared of 0.63), branching (*R*-squared of 0.63), and length (*R*-squared of 0.80). This method outperformed the same model trained only on original images, particularly in distinguishing LSCD with an AUC of 0.867. The results suggest that supplementing training data with latent diffusion-generated images can effectively enhance model performance.

## Privacy and continual learning in AI

Verma et al. explored privacy-preserving methods in continual learning for medical image classification, focusing on retinal disease detection from OCT images and histology-based colon cancer classification. Their study revealed that Brain-Inspired Replay (BIR) excelled in retinal disease classification with notable accuracy, while Efficient Feature Transformations (EFT) were most accurate for colon cancer detection. They found that these methods, though slightly outperformed by joint retraining models, offer significant benefits for long-term clinical use by reducing catastrophic forgetting and enabling ongoing model updates without compromising patient privacy.

## Conclusion

The breadth of this Research Topic, with its collection of varied and insightful studies, underscores the remarkable potential of big data and AI within ophthalmology. The contributions from the authors, with their innovative algorithms and forward-thinking perspectives, hold significant promise for transforming the fabric of eye care. These studies serve as cornerstones upon which future collaborative endeavors can be built, driving the advancement of ophthalmological practices into a new era. As we stand on the precipice of this technological revolution, the integration of these AI-driven tools and methodologies heralds a progressive shift toward enhanced patient care.

## Author contributions

YT: Conceptualization, Writing – original draft, Writing – review & editing. TR: Writing – review & editing, Conceptualization. DT: Writing – review & editing. Y-TH: Writing – review & editing. T-iK: Writing – review & editing.

